# Transcatheter Aortic Valve Replacement Complicated by Aorto–Right Ventricular Fistula Repaired Via Valve-in-Valve Replacement

**DOI:** 10.1016/j.jaccas.2023.102165

**Published:** 2023-12-15

**Authors:** Omar M. Masarweh, Anusha Ghaffar, Stephanie Lopez-Orizondo, Olga Karasik, Jooby John

**Affiliations:** a University of Central Florida/HCA Healthcare GME, Greater Orlando, Florida, USA; b Department of Internal Medicine, University of Central Florida College of Medicine, Orlando, Florida, USA

**Keywords:** complication, TAVR, valve implantation

## Abstract

A 59-year-old man with a history of severe aortic stenosis with transcatheter aortic valve replacement (TAVR) presented with worsening heart failure. Echocardiography showed a fistula between the aorta and the right ventricle with mild to moderate paravalvular regurgitation. He underwent a valve-in-valve TAVR with symptomatic improvement and decreased flow through the fistula.

## Graphical abstract

**Figure:**
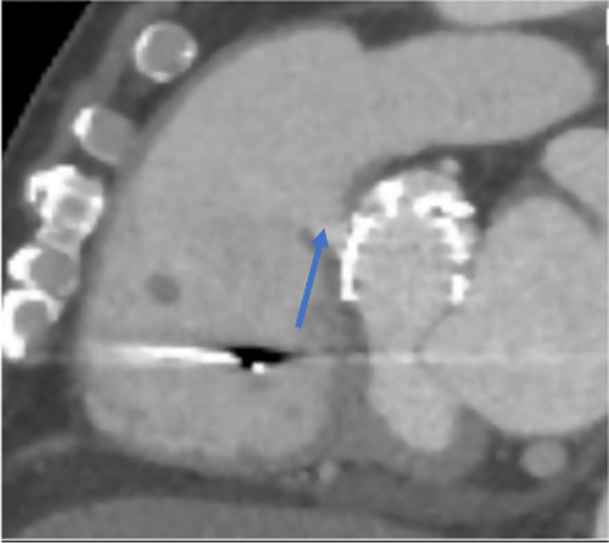

## History of Presentation

A 59-year-old man presented with signs and symptoms of right- and left-sided heart failure with NYHA functional class IV symptoms for 1 month, with worsening generalized swelling, shortness of breath, and abdominal distention.

**Learning Objectives**

• To be able to make a differential diagnosis of fistula formation with echocardiography and cardiac CT.
• To identify patients who may benefit from fistula repair and identify available repair techniques.

## Past Medical History

His medical history included severe aortic stenosis status post transcatheter aortic valve replacement (TAVR) 3 years earlier, heart failure with recovered ejection fraction (30%-35% to 60%) status post biventricular implanted cardioverter-defibrillator placement, paroxysmal atrial fibrillation, type 2 diabetes mellitus, and chronic kidney disease stage 3B.

## Differential Diagnosis

The differential diagnosis included worsening of pre-existing heart failure, worsening aortic stenosis, and paravalvular regurgitation.

## Investigations

**Figure 1:**
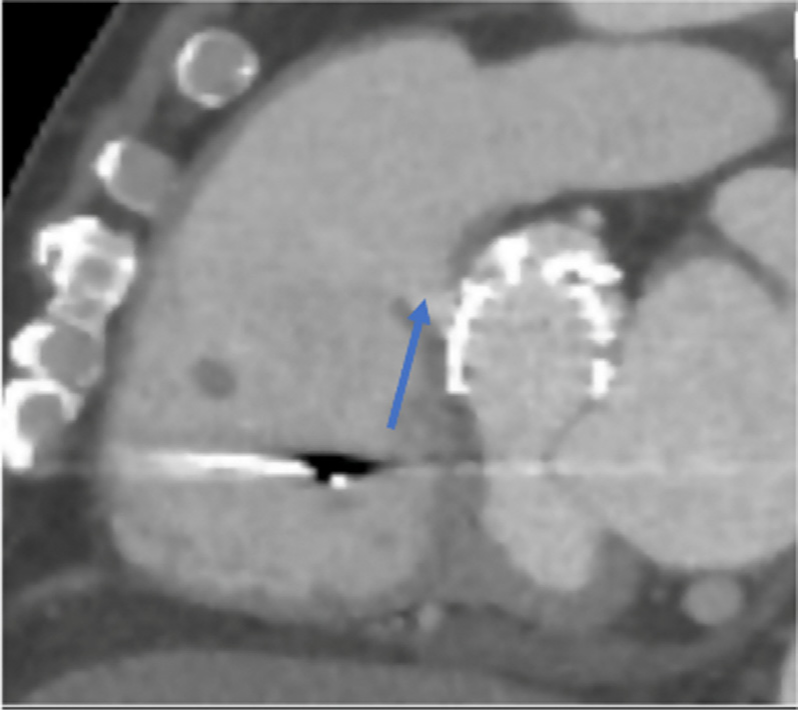
Cardiac Computed Tomography in the Sagittal View The image shows the fistula connecting the aorta near the right sinus to the right ventricle (arrow).

On arrival, he was hemodynamically stable, with NYHA functional class IV heart failure symptoms, elevated jugular venous pressures, anasarca, and ascites. Laboratory data showed B-type natriuretic peptide (BNP) at 2,955 pg/mL (reference range <100 pg/mL). A transthoracic echocardiogram (TTE) revealed an aortic valve area of 1.3 cm^2^, a peak velocity of 2 m/s, an ejection fraction of 60%, mild to moderate aortic insufficiency, and a communication between the aorta and the right ventricle (Video 1). A transesophageal echocardiogram (TEE) showed a communication between a sinus adjoining the right coronary cusp and the right ventricle (Video 2). Cardiac computed tomography (CT) measured the aortic annulus at 32 × 30.4 mm, with a diameter of 772 mm^2^, and visualized the fistula (Figure 1). Left- and right-sided heart catheterization showed oxygen step-up with a right ventricular (RV) saturation of 71% and a pulmonary artery saturation of 82%.

## Management

**Figure 2:**
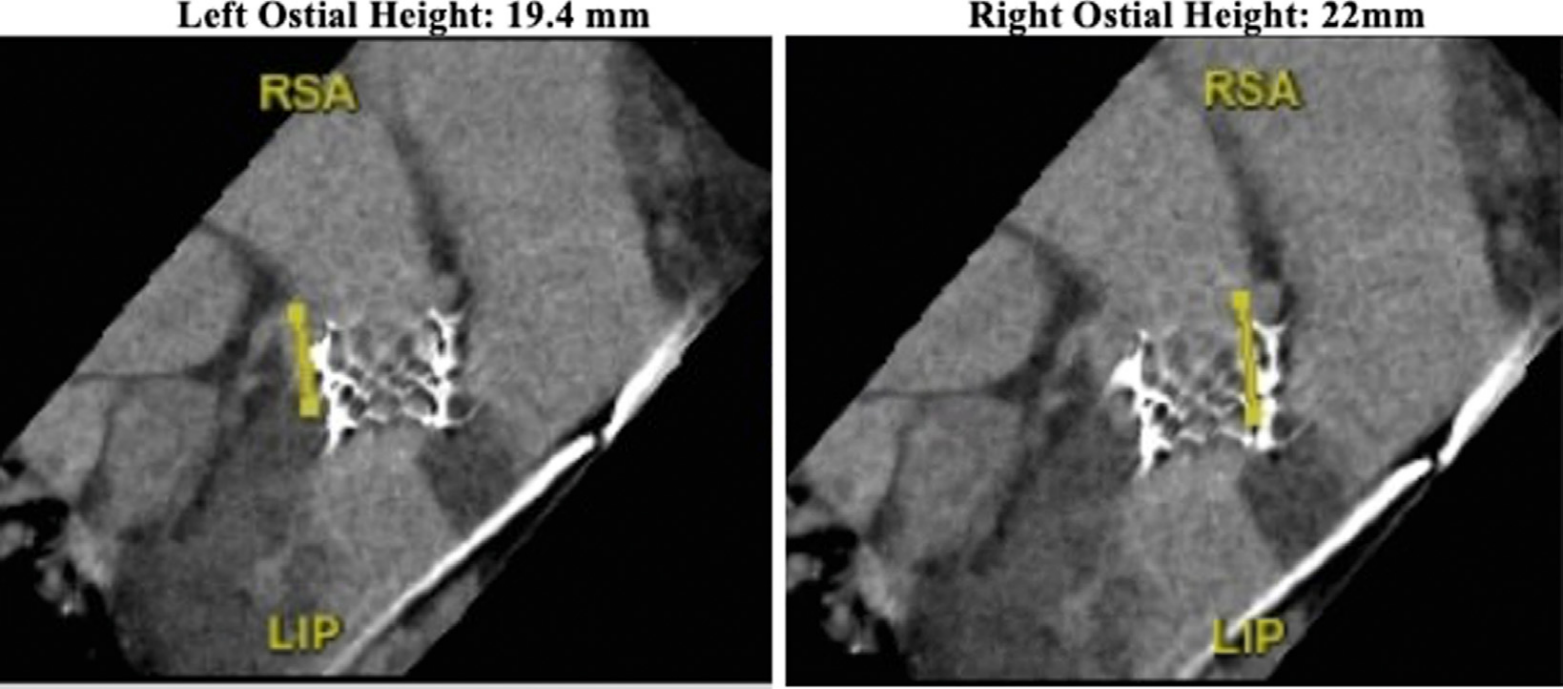
Computed Tomography Showing Right and Left Coronary Heights Relative to Previous Transcatheter Aortic Valve Replacement

A comprehensive heart team discussion ensued. Because the patient was a poor surgical candidate and had both aortic regurgitation and an aorto-RV fistula, it was believed that we could potentially accomplish treatment of both the bioprosthetic regurgitation and the fistula with placement of a second TAVR valve, thereby ideally sealing off the fistula and correcting the aortic regurgitation. The patient then underwent a valve-in-valve TAVR with placement of a Evolut Pro Plus 34-mm TAVR valve (Medtronic) over the pre-existing Edwards Sapien valve (Edwards Lifesciences). By using the previous valve as a landmark, we were able to land the new valve just below the prosthesis. Preoperative CT planning showed coronary heights to be 19.4 mm on the left and 22 mm on the right. Deploying the second valve just below the pre-existing valve ensured that the coronary ostia would not be occluded (Figure 2). The valve was deployed under rapid pacing and post-dilated. After this, the fistulous connection was found to be still patent, but flow had dramatically reduced (Videos 3 and 4).

## Discussion

As the number of TAVR procedures performed continues to increase, we are likely to encounter infrequent complications. A comprehensive search of the published literature revealed 14 cases of aorto-RV fistula formation following TAVR. However, this appears to be the first reported case of a fistula repaired using a valve-in-valve approach.^1^

Possible mechanisms of fistula formation following TAVR may be a contained annular rupture at the time of valve deployment with drainage into the right ventricle or trauma from displacement of heavily calcified tissue near the annulus of the aorta.^2^ In fact, annular rupture has almost exclusively been seen with balloon-expandable valves rather than self-expanding valves, unless post-dilatation is used for the latter.^3^ Other considerations predisposing to annular rupture include the size of the aortic valve annulus (<20 mm) or a narrow aortic root. However, despite these considerations, there may be undetermined factors that contribute to annular rupture.

Annular rupture and formation of fistulas can develop in 4 anatomical areas: intra-annular, subannular, supra-annular, or combined. Once a rupture has occurred, clinical manifestations usually appear abruptly, evident by the patient’s hemodynamics. However, presentation can be delayed for a few hours or more if the injury is small. Some fistulas may remain silent for years, as in our patient, and can even remain unrecognized.^3^ If left untreated, these fistulas can cause biventricular failure, pulmonary hypertension, and death.^2^ RV failure stems from increased preload from left ventricular to RV shunting and increased RV afterload from pulmonary hypertension.

When deciding which patient should undergo repair or conservation management, the ratio of pulmonary flow to systemic flow (Qp/Qs ratio) has been hypothesized as a possible indicator of severity.^4^ In a review of the previous 14 known cases of aorto-RV fistula following TAVR, most asymptomatic patients had a Qp/Qs ratio <1.80, but in the 9-month to 4-year follow-up period, 3 of the 7 asymptomatic patients died; the other 4 patients remained asymptomatic.^4^ It is evident that it is difficult to assess which patients’ fistulas will progress to the point that intervention is necessary because the timing of clinical presentation may vary depending on the chronicity and size of the fistula, as well as hemodynamic changes such as pulmonary hypertension or RV failure. Deciding to intervene on a fistula requires proper and diligent planning. Percutaneous coiling, atrial septal defect or ventricular septal defect occluders, and surgical repair have all been reported, with various success rates. When determining which intervention would be appropriate, assessing coronary heights should be considered because undergoing a valve-in-valve replacement may obstruct the coronary ostia.

The decision to intervene in this patient came after a comprehensive heart team discussion. The team decided that given the evidence of significant right-sided heart failure, he would benefit from intervention as opposed to medical therapy. There are insufficient long-term outcome data on intervention vs conservative management in asymptomatic and symptomatic patients. The decision whether to intervene in such complex patients would best be managed by a multidisciplinary heart team, and these patients are ideally observed closely and reassessed frequently with echocardiography and advanced cardiac imaging.

There still remain numerous unanswered questions when it comes to aorto-RV fistulas. How does medical management compare with surgical or percutaneous repair in terms of overall survival and symptoms? What is the longevity of these repaired fistulas? Unfortunately, there are no published data on the long-term prognosis and development of further complications in patients with repaired aorto-RV fistulas.

## Follow-Up

Two weeks after the procedure, the patient was seen in clinic and continued to improve, with NYHA functional class I to II symptoms. His repeat BNP value was 358 pg/dL. Our plan is to observe the patient clinically, and if there is clinical decline, we will consider percutaneous coiling or surgical patch repair.

## Conclusions

Aorto-RV fistula formation following a TAVR procedure is a rare and potentially life-threatening complication that needs to be on the radar of all clinicians. With so few cases reported and no consensus guidelines available for management, we are all better off with more data.

## Funding Support and Author Disclosures

This research was supported (in whole or in part) by the HCA Healthcare and/or an HCA Healthcare affiliated entity. The views expressed in this publication represent those of the author(s) and do not necessarily represent the official views of HCA Healthcare or any of its affiliated entities. The authors have reported that they have no relationships relevant to the contents of this paper to disclose.

## Appendix

Video 1 Preoperative Transthoracic Echocardiogram in the Parasternal Long-Axis View Showing Mild to Moderate Aortic Regurgitation

Video 2 Preoperative Transesophageal Echocardiogram Showing Flow From the Aorta Near the Right Coronary Sinus Into the Right Ventricle

Video 3 Postoperative Transesophageal Echocardiogram Showing Reduced Flow Through the Fistula

Video 4 Postoperative Transthoracic Echocardiogram in the Parasternal Long-Axis View Showing Reduced Flow Through the Fistula
